# Therapeutic Options in Refractory Evans Syndrome: A Case Report

**DOI:** 10.7759/cureus.32635

**Published:** 2022-12-17

**Authors:** Nina Jancar, Filipa Sousa Gonçalves, Catarina Jacinto Correia, José Duro, Patrício Aguiar

**Affiliations:** 1 Internal Medicine, Hospital de Santa Maria, Centro Hospitalar Universitário Lisboa Norte, Lisbon, PRT; 2 Transfusion Medicine, Hospital de Santa Maria, Centro Hospitalar Universitário Lisboa Norte, Lisbon, PRT

**Keywords:** immunosuppression, infectious complications, bicytopenia, idiopathic, evans’ syndrome

## Abstract

Evans syndrome is a rare autoimmune disease, characterized by at least two immune cytopenias, most frequently anemia and thrombocytopenia and rarely immune neutropenia. It has a variable clinical presentation and is rarely diagnosed in adults. It can be idiopathic or secondary to lymphoproliferative disease, infections, autoimmune diseases, drugs, and immunodeficiencies in about 50% of cases. It is characterized by a chronic, relapsing, potentially fatal course due to its hemorrhagic complications as well as complications associated with the long-term immunosuppressive treatment required to control the disease, such as infectious diseases, and cardiovascular and renal complications. Its prognosis depends on the underlying cause. Because of its rarity, the treatment is empirical, based mostly on case series and recommendations for the treatment of other immune cytopenias. The underlying disease and demographic characteristics also play an important role in choosing the treatment, which should be adapted individually to each patient. We present a case of an elderly patient with idiopathic autoimmune hemolytic anemia and thrombocytopenia, refractory to various treatment options.

## Introduction

Evans syndrome (ES) is a chronic, rare autoimmune disease characterized by the coexistence of autoimmune hemolytic anemia (AIHA) and thrombocytopenia, or less frequently, immune neutropenia [1,2-4]. It is rarely observed in adults, with an estimated incidence of 1.8/million person-years and an annual prevalence of 21.3/million persons; it can be idiopathic or, in about 50% of cases, secondary to lymphoproliferative disease, autoimmune disease, infectious diseases, drugs and primary immunodeficiencies [2,3]. Therefore, it is imperative to identify any underlying cause, in order to initiate the appropriate treatment and define the patient’s prognosis [2]. Furthermore, it has a chronic, relapsing course, sometimes needing various therapeutic lines to achieve remission. We present a clinical case of an elderly patient with idiopathic refractory ES. This clinical case was presented in the form of a poster at the 27º Congresso Nacional de Medicina Interna (Portugal, 2-5 October 2021).

## Case presentation

We report a case of an 80-year-old male patient with a medical history of essential arterial hypertension and chronic ulcers of the lower limbs secondary to deep vein insufficiency. He did not report any other relevant medical history or previous surgeries and did not take any medication due to medication non-adherence. The patient sought medical attention after an accidental fall from a standing height, resulting in cranioencephalic trauma and altered mental status. On physical examination, altered mental status was denoted-the patient was alert but disoriented to time and space and agitated. Multiple cutaneous hematomas of the limbs and torso, gingival bleeding, and systolic murmur (grade II/VI) were also observed. No focal neurological deficit was noted, and the rest of the examination was unremarkable.

**Figure 1:**
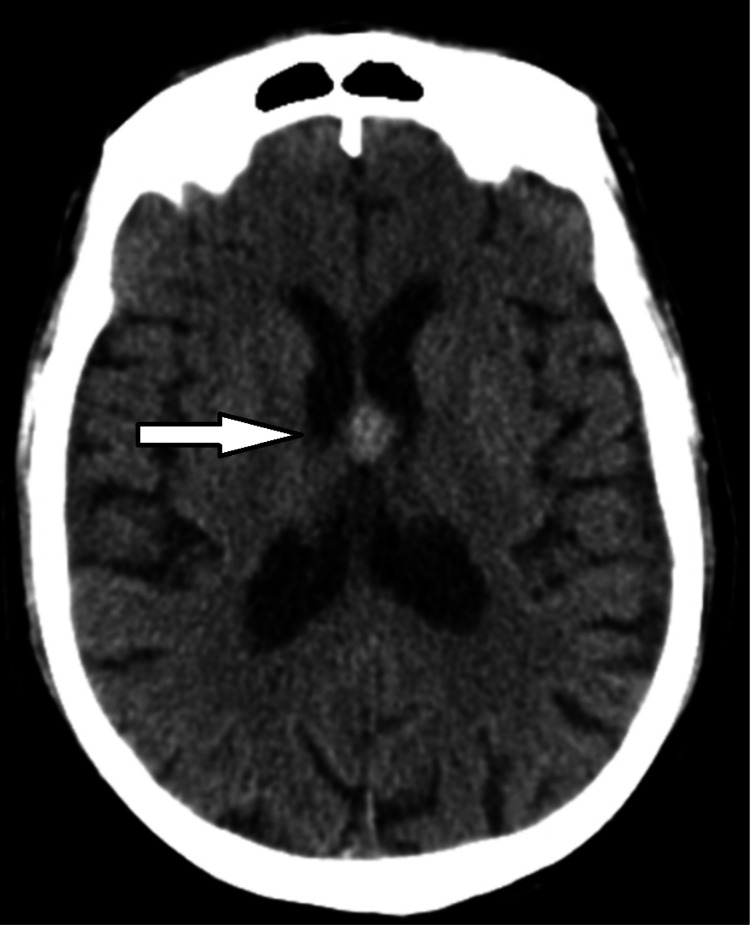
Cranial computed tomography (CT) showing a hyperdense lesion, localized to the inferior pole of the septum pellucidum, suggestive of recent hemorrhage

Laboratory investigations showed normocytic anemia (hemoglobin 6.2 g/dL) and thrombocytopenia (1000/uL), a positive direct Coombs test, as well as the presence of antiplatelet antibodies. The peripheral blood smear was unremarkable. A cranial computed tomography (CT) scan showed a hyperdense lesion localized to the inferior pole of the septum pellucidum, suggestive of a recent hemorrhage (Figure 1).

ES was suspected, and further diagnostic tests were performed to determine the etiology. Autoimmune disease profile, infective serologies most frequently associated with ES (human immunodeficiency virus (HIV), hepatitis C, hepatitis B, cytomegalovirus (CMV), Epstein-Barr virus (EBV), *Mycoplasma pneumoniae*), cryoglobulins, and cold agglutinins were all negative, but there was complement consumption. Immunofixation, myelogram, and thoracic, abdominal, and pelvic CT scans were unremarkable, leading to the idiopathic ES diagnosis.

The patient was started on intravenous immunoglobulin (0.4 g/Kg/day, five days) and intravenous methylprednisolone pulse therapy (1 g/day, five days) as well as platelet transfusions without response and maintained severe thrombocytopenia (4000/uL platelets), complicated by upper gastrointestinal tract bleeding, presenting as melena. Upper endoscopy was performed and showed erythematous petechial gastropathy. No significant findings were identified on the histological examination of biopsies.

In light of the life-threatening hemorrhage, despite the initial therapy, vincristine and high-dose prednisolone were started, yielding stabilization of bleeding diathesis, without a significant increase in platelet count or hemoglobin level. Because of the refractoriness to vincristine, azathioprine was added to high-dose prednisolone, resulting in an increase in hemoglobin and platelet levels. After an attempt to taper prednisolone, there was a relapse of thrombocytopenia. A fourth-line therapy with eltrombopag, in conjunction with prednisolone, was started after consultation with a hematologist, with a good response and normalization of platelet count.

During the hospitalization, various infectious complications were diagnosed, including nosocomial pneumonia (for which the patient was treated with piperacillin/tazobactam), carbapenemase-resistant *Klebsiella pneumoniae* bacteriemia (for which the patient was treated with meropenem) and herpes simplex virus type 1 (HSV1) infection.

At discharge, the patient was medicated with eltrombopag, prednisolone, and methotrexate as a corticoid-sparing agent, given the history of corticoid dependency, maintaining a long-term normal platelet count and stable hemoglobin.

## Discussion

ES is a chronic autoimmune disease characterized by two or more cytopenias, the most frequently warm antibody AIHA, and immune thrombocytopenia (ITP). Immune neutropenia is present in 4-15% of all cases [1,5-6]. It is a heterogeneous disease with variable clinical presentation, the natural course of which is characterized by remissions and exacerbations [2].

It is rarely diagnosed in adults (according to some reports, it is diagnosed in about 5% of all patients diagnosed with ITP or AIHA), and the estimated incidence is 1 to 9 cases per million people per year [5,6]. It is most frequently diagnosed in the fifth decade and is more prevalent in females (3:2 female to male ratio) [1,5]. The patient we present in our case report was male and was 80 years old at the onset.

The clinical presentation is variable and depends on the affected blood cell lines; patients frequently present with fatigue, dyspnea, and dizziness, symptoms associated with hemolytic anemia, and bleeding manifestations related to thrombocytopenia [3,5]. Although our patient had severe anemia and thrombocytopenia at admission, he did not have any complaint/symptom before the accidental fall and subsequent cranioencephalic trauma for which he sought medical attention. As far as physical examination goes, he presented with diffuse hematomas and oral cavity hemorrhage associated with severe thrombocytopenia, as well as mental confusion resulting from the intracranial hemorrhage. During the hospitalization, upper gastrointestinal tract bleeding without hemodynamic instability in the form of melena was detected. Other signs associated with ES, such as jaundice, choluria, and splenomegaly, were absent [5,7].

The initial evaluation of a patient with a suspected ES should include a complete blood count, hemolysis parameters, Coombs test, and platelet antibody dosing, as well as peripheral blood smear (which will show no or few schistocytes, some spherocytes, and poikilocytosis), which will, along with renal function evaluation, help differentiate ES from thrombotic microangiopathies (TMA)/thrombotic thrombocytopenic purpura (TTP) [3,4].

It is classified as primary (idiopathic) and secondary ES, which is frequently associated with autoimmune diseases (systemic lupus erythematosus, antiphospholipid syndrome, Sjogren syndrome, Hashimoto thyroiditis, inflammatory bowel disease), infections (HIV, hepatitis C, EBV, CMV, severe acute respiratory syndrome coronavirus 2 (SARS-CoV2)), immunodeficiencies (Common variable immunodeficiency, IgA deficiency), lymphoproliferative diseases (non-Hodgkin lymphoma and chronic lymphocytic leukemia) and other hematologic disorders (monoclonal gammopathy of undetermined significance (MGUS), autoimmune lymphoproliferative syndrome (ALPS), chronic myelomonocytic leukemia, idiopathic CD4 lymphocytopenia), as well as solid tumors (thymoma/ovarian dermoid cyst/carcinoma), and other conditions (pregnancy, post allogeneic bone marrow transplantation, Kabuki syndrome) [1,8,9].

Since ES has an identifiable cause in about 20-50% of cases, extensive evaluation of underlying etiologies is essential, as they can influence the treatment and prognosis [2,5,7-8]. Viral serologies (HIV, hepatitis C, and B, *Mycoplasma*, CMV, and EBV), autoimmune profile (antinuclear and anti-dsDNA antibodies), immunoglobulin concentrations, and bone marrow study, as well as imaging studies (thoracic, abdominal, and pelvic CT scan), can help determine underlying conditions and were all unremarkable in our patient, which led to the diagnosis of primary (idiopathic) ES.

Due to its rarity, the treatment of ES is empirical, and the recommendations are based primarily on case series, as well as recommendations and guidelines for the treatment of ITP and AIHA.

Therapy with high-dose corticosteroids remains the therapy of choice, with initial response rates as high as 80%. However, about 60-76% of patients will need second-line treatment, either due to failure to achieve initial response (about 20%) or due to relapse/corticosteroid dependency [7]. Therapeutic options for the treatment of relapses or refractory ES consist of rituximab, splenectomy, immunosuppressive drugs (cyclophosphamide, azathioprine, ciclosporin, mycophenolate, and vincristine), as well as thrombopoietin receptor agonists [4,9].

Our patient presented with life-threatening hemorrhage (intracranial hemorrhage and gastrointestinal bleeding) due to severe thrombocytopenia and was therefore started on methylprednisolone pulses, immunoglobulin, and platelet transfusions, followed by vincristine infusion. After achieving hemostasis, the platelet count and hemoglobin remained low. Therefore, azathioprine and high-dose prednisolone were started, with another relapse and the need to start a fourth-line therapy with eltrombopag, prednisolone, and corticosteroid-sparing agent to achieve long-term stability of the disease. Rituximab is a monoclonal, chimeric antibody against CD20 used for the treatment of various hematologic and autoimmune diseases, often used as a second-line therapy for ES. It causes depletion of B cells, leading to neutropenia and, more importantly, hypogammaglobulinemia, which increases the risk for severe infections, including infections with opportunistic agents [10]. The risk for serious infectious complications, associated with higher mortality, is more significant in elderly and frail patients due to their comorbidities, immunosenescence, and alterations in pharmacokinetics, which presents a challenge in treating patients with autoimmune diseases [11]. Given the multiple relapses, suggesting the need for long-term therapy in our patient, we opted against treatment with rituximab, given the complications, more often documented in elderly and fragile patients.

As is evident from our case report, the disease itself is associated with various complications, the most relevant being infections, bleeding, massive hemolysis, and thrombotic events, and we would like to emphasize the importance of not only a focused immunosuppressive therapy but also supportive care and management of the complications.

Because of the complications mentioned above, ES remains a disease with a high mortality rate (20-24%), especially in secondary ES, associated with hematologic malignancies [7].

## Conclusions

ES is a chronic autoimmune disease characterized by at least two cytopenias and is rarely diagnosed in adults, especially in the elderly. It is idiopathic in about 50% of cases. Although long-term remission with first-line therapy is rare, multiple refractoriness is not frequently described.

In our case, multiple relapses were documented, and various therapy lines were necessary to achieve remission. Prolonged immunosuppression therapy resulted in various infectious complications, causing a negative impact on the morbidity of our patient.

In conclusion, prompt diagnosis of ES and exclusion of underlying causes is essential since it can alter de treatment course and prognosis. Furthermore, immunosuppressive treatment is often challenging due to its many possible adverse effects, which are more frequent in elderly and frail patients and should be adapted to each patient and their comorbidities individually.
